# An upper bound for the *Z*-spectral radius of adjacency tensors

**DOI:** 10.1186/s13660-018-1672-4

**Published:** 2018-04-06

**Authors:** Zhi-Yong Wu, Jun He, Yan-Min Liu, Jun-Kang Tian

**Affiliations:** School of Mathematics, Zunyi Normal College, Zunyi, P.R. China

**Keywords:** 15A18, 15A69, 65F15, 65F10, Hypergraph, *Z*-eigenvalue, Bound, Nonnegative tensor

## Abstract

Let $\mathcal{H}$ be a *k*-uniform hypergraph on *n* vertices with degree sequence $\Delta=d_{1} \geq\cdots\geq d_{n}=\delta$. In this paper, in terms of degree $d_{i}$, we give a new upper bound for the *Z*-spectral radius of the adjacency tensor of $\mathcal{H}$. Some examples are given to show the efficiency of the bound.

## Introduction

Let $\mathcal{A}=(a_{i_{1} i_{2} \cdots i_{m} } )$ be an *m*th order *n*-dimensional real square tensor, *x* be a real *n*-vector. Then we define the following real *n*-vector:

$$\mathcal{A}x^{m-1}= \Biggl(\sum_{i_{2} , \ldots,i_{m} = 1}^{n} {a_{ii_{2} \cdots i_{m} } }x_{i_{2}} \cdots x_{i_{m}} \Biggr)_{1\leq i \leq n}, \qquad x^{[m-1]}= \bigl(x_{i}^{m-1}\bigr)_{1\leq i \leq n}. $$

If there exist a real vector *x* and a real number *λ* such that

$$\mathcal{A}x^{m-1}=\lambda x^{[m-1]}, $$

then *λ* is called an H-eigenvalue of $\mathcal{A}$ and *x* is called an eigenvector of $\mathcal{A}$ associated with *λ* [1, 2]. If there exist a real vector *x* and a real number *λ* such that

$$\mathcal{A}x^{m-1}=\lambda x,\qquad x^{T}x=1, $$

then *λ* is called a *Z*-eigenvalue of $\mathcal{A}$ and *x* is called an eigenvector of $\mathcal{A}$ associated with *λ*. You can see more about the eigenvalues of tensors in [3–7].

Let $\mathcal{H}$ be a hypergraph with a vertex set $V (\mathcal{H}) $ and an edge set $E(\mathcal{H})= \{e_{1},e_{2},\ldots,e_{t}\}$. If every edge of $\mathcal{H}$ contains exactly *k* distinct vertices, then $\mathcal{H}$ is called a *k*-uniform hypergraph. The degree of a vertex *i* in $\mathcal{H}$ is the number of edges incident with *i*, denoted by $d_{i}$. If $d_{i} = d$ for any $i \in V (\mathcal {H})$, then the hypergraph $\mathcal{H}$ is called a regular hypergraph. Recently, the spectral radii of hypergraphs have been studied in [8, 9].

Let $\{i_{1},\ldots,i_{k}\} \in E(\mathcal{H})$ mean that there is an edge containing *k* distinct vertices $i_{1}, \ldots, i_{k}$. Then the adjacency tensor $\mathcal{A}(\mathcal{H})=(a_{i_{1}\cdots i_{k}})$ of a hypergraph $\mathcal{H}$ is a *k*th order *n*-dimensional tensor with entries:

$$a_{i_{1} \cdots i_{k} }= \textstyle\begin{cases} \frac{1}{(k - 1)!}, & \mbox{if } \{i_{1},\ldots,i_{k}\} \in E(\mathcal {H}),\\ 0, & \mbox{otherwise}. \end{cases} $$

Let $D(\mathcal{H}) = \operatorname{diag}(d_{1}, d_{2}, \ldots, d_{n})$ be the degree diagonal tensor of the graph $\mathcal{H}$. Then the tensor $Q(\mathcal {H}) = D(\mathcal{H})+ \mathcal{A}(\mathcal{H})$ is called the signless Laplacian tensor of the hypergraph $\mathcal{H}$. The largest modulus of the *Z*-eigenvalues of the adjacency tensor $\mathcal{A}(\mathcal{H})$ is denoted by $\rho_{Z}(\mathcal{H})$, which is called the *Z*-spectral radius of the adjacency tensor $\mathcal{A}(\mathcal{H})$.

For a *k*-uniform hypergraph $\mathcal{H}$, let $\Delta=d_{1} \geq\cdots \geq d_{n}=\delta$ be the degree sequence of the hypergraph $\mathcal{H}$. In 2013, Xie and Chang [8] presented the following upper bound for the largest *Z*-eigenvalues $\rho_{Z} (\mathcal {H})$ of adjacency tensors:

1 $$ \rho_{Z} (\mathcal{H}) \le\Delta. $$

In this paper, we give a new upper bounds in terms of degree $d_{i}$ for the *Z*-spectral radius of hypergraphs, which improves the bound as shown in (1). Then we give some examples to compare these bounds for *Z*-spectral radius of hypergraphs.

## Preliminaries

Some basic definitions and useful results are listed as follows.

### Definition 2.1

([10])

The tensor $\mathcal {A}$ is called reducible if there exists a nonempty proper index subset $\mathbb{J} \subset\{ 1,2,\ldots ,n \}$ such that $a_{i_{1},i_{2},\ldots,i_{m}} = 0$, $\forall i_{1} \in\mathbb{J}$, $\forall i_{2}, \ldots, i_{m} \notin\mathbb{J}$. If $\mathcal{A}$ is not reducible, then we call $\mathcal{A}$ to be irreducible.

### Definition 2.2

Let $\mathcal{A}$ be an m-order and n-dimensional tensor. We define $\sigma(\mathcal{A})$ the *Z*-spectrum of $\mathcal{A}$ by the set of all *Z*-eigenvalues of $\mathcal{A}$. Assume $\sigma(\mathcal{A})\neq \emptyset$, then the *Z*-spectral radius of $\mathcal{A}$ is denoted by

$$\rho_{Z} (\mathcal{A})= \max\bigl\{ |\lambda|: \lambda\in\sigma( \mathcal{A}) \bigr\} . $$

The concept of *weakly symmetric* was first introduced and used by Chang, Pearson, and Zhang [11] in order to study the following Perron–Frobenius theorem for the *Z*-eigenvalue of nonnegative tensors.

### Lemma 2.1

([11])

*Let* $\mathcal{A}=(a_{i_{1}i_{2} \cdots i_{m}})$ *be a weakly symmetric nonnegative tensor*, *then the spectral radius* $\rho _{Z} (\mathcal{A})$ *is a positive* *Z*-*eigenvalue with a nonnegative* *Z*-*eigenvector* *x*. *Furthermore*, *if* $\mathcal{A}$ *is irreducible*, *x* *is positive*.

$|\mathcal{A}|$ means that $(|\mathcal{A}|)_{i_{1} \cdots i_{m}}=|a_{i_{1} \cdots i_{m}}|$. Two useful lemmas are given as follows.

### Lemma 2.2

*Let* $\mathcal{A}$ *and* $\mathcal{B}$ *be two weakly symmetric and irreducible tensors of order* *m* *and dimension* *n*. *If* $\mathcal{B}$ *and* $\mathcal{B} - |\mathcal{A}|$ *are nonnegative*, *then* $\rho_{Z}(\mathcal{B}) \geq\rho_{Z}(|\mathcal{A}|)$.

### Proof

Let *y* be the eigenvector associated with *β*, where *β* is a *Z*-eigenvalue of $\mathcal{A}$. Then we can get

$$\vert \beta \vert \vert y \vert = \bigl\vert \mathcal{A}y^{[m-1]} \bigr\vert \leq \vert \mathcal{A} \vert \bigl\vert y^{[m-1]} \bigr\vert \leq \mathcal{B} \bigl\vert y^{[m-1]} \bigr\vert . $$

By Theorem 4.7 of [11], we have

$$\rho_{Z} (\mathcal{B}) = \max_{y \ge0} \min _{ \vert {y_{i} } \vert > 0} \frac{{ ( {\mathcal{B} \vert y \vert ^{[m - 1]} } )_{i} }}{{ \vert {y_{i} } \vert }} \ge\min_{ \vert {y_{i} } \vert > 0} \frac {{ ( {\mathcal{B} \vert y \vert ^{[m - 1]} } )_{i} }}{{ \vert {y_{i} } \vert }} \ge|\beta|. $$

Then

$$\rho_{Z}(\mathcal{B}) \geq\rho_{Z}\bigl( \vert \mathcal{A} \vert \bigr). $$

 □

### Lemma 2.3

*Let* $\{\mathcal{A}_{k}\}$ *be a sequence of nonnegative*, *weakly symmetric tensors of order* *m* *and dimension* *n*, *and* $\mathcal{A}_{k} - \mathcal{A}_{k+1}$ *be nonnegative for each positive integer* *k*. *Then*

$$\lim_{k \to\infty} \rho_{Z} ( {\mathcal{A}_{k} } ) = \rho _{Z} \Bigl( { \lim_{k \to\infty} \mathcal{A}_{k} } \Bigr). $$

### Proof

Let $\mathcal{A} = \lim_{k \to\infty} \mathcal{A}_{k} $. Since $\mathcal{A}_{k} - \mathcal{A}_{k+1}$ is nonnegative, by Lemma 2.2, we know that $\{ \rho_{Z} ( {\mathcal{A}_{k} } )\}$ is a monotone decreasing sequence with a lower bound $\rho_{Z} (\mathcal{A})$. So $\lim_{k \to\infty} \mathcal{A}_{k}$ exists and

$$\lambda= \lim_{k \to\infty} \rho_{Z} ( { \mathcal{A}_{k} } )\geq\rho_{Z}(\mathcal{A}). $$

Since $\{\mathcal{A}_{k}\}$ is nonnegative, weakly symmetric, then there exists a nonnegative vector $x^{(k)}$ such that $\mathcal {A}_{k}(x^{(k)})^{m-1}=\rho_{Z} ( {\mathcal{A}_{k} } )x^{(k)}$ and $(x^{(k)})^{T}x^{(k)} = 1$. Then $\{x^{(k)}\}$ is a bounded sequence, it has a convergent subsequence $\{y_{t} \}$. Suppose that $y = \lim_{k \to\infty} y_{t}$. By $\mathcal{A}_{k}y_{t}^{m-1}=\rho_{Z} ( {\mathcal{A}_{k} } )y_{t}$, we get $\mathcal{A}y^{m-1}=\lambda y$. So *λ* is an eigenvalue of $\mathcal{A}$. Since $\lambda\leq\rho_{Z}(\mathcal{A})$, we have $\rho _{Z}(\mathcal{A}) = \lambda$. □

## The *Z*-spectral radius of tensors and hypergraphs

In this section, let $r_{i}(\mathcal{A})=\sum_{i_{2},\ldots,i_{m} = 1}^{n} |a_{ii_{2}\cdots i_{m}}|-|a_{ii\cdots i}|$, we give some bounds on the *Z*-spectral radius of tensors and hypergraphs.

### Theorem 3.1

*Let* $\mathcal{A}$ *be weakly symmetric nonnegative tensors of order* *m* *and dimension n*. *Then*

$$\rho_{Z}(\mathcal{A})\leq \max_{a_{i_{1} \cdots i_{m} } \ne0} \Biggl\{ { \prod_{j = 1}^{m} {r_{i_{j} }^{\frac{1}{m}} (\mathcal{A})} } \Biggr\} . $$

### Proof

Case 1. If $\mathcal{A}$ is irreducible, by Lemma 2.1, let $u = (u_{i})$ be the positive eigenvector associated with the largest *Z*-eigenvalues $\rho_{Z} (\mathcal{A})$ of $\mathcal{A}$. Then

$$\mathcal{A}u^{m-1}=\rho_{Z}(\mathcal{A})u. $$

Let ${u_{\alpha}} = \max \{ { {u_{i_{1} } } \cdots {u_{i_{m} } } :a_{i_{1} \cdots i_{m} } \ne0,1 \le i_{1} , \ldots,i_{m} \le n} \}$, then

2 $$\begin{aligned} \begin{aligned}[b] \rho_{Z}(\mathcal{A})u_{i}^{2} &=\sum _{i_{2} , \ldots,i_{m} = 1}^{n} {a_{ii_{2} \cdots i_{m} } }u_{i} u_{i_{2}} \cdots u_{i_{m}} \\ &=\sum_{a_{ii_{2} \cdots i_{k} }\neq0} {a_{ii_{2} \cdots i_{k} } }u_{i} u_{i_{2}} \cdots u_{i_{m}} \\ &\leq r_{i}(\mathcal{A}) {u_{\alpha}} . \end{aligned} \end{aligned}$$

Suppose that ${u_{\alpha}} = {u_{j_{1} } } \cdots {u_{j_{m} } } $. Then, from (2), we can get

$$\begin{aligned}& \rho_{Z}(\mathcal{A})u_{j_{1}}^{2} \leq r_{j_{1}}(\mathcal{A}) {u_{\alpha}}, \\& \vdots \\& \rho_{Z}(\mathcal{A})u_{j_{m}}^{2} \leq r_{j_{m}}(\mathcal{A}) {u_{\alpha}}. \end{aligned}$$

Then, by $u_{\alpha}^{m} \leq u_{\alpha}^{2}$, we have

$$\prod_{l = 1}^{m} {\rho_{Z}^{m}( \mathcal{A})u_{j_{l}}^{2}} \leq u_{\alpha}^{m} \prod_{l = 1}^{m} {r_{i_{l} } ( \mathcal{A})} \leq u_{\alpha}^{2} \prod _{l = 1}^{m} {r_{i_{l} } (\mathcal{A})}. $$

Therefore,

$$\rho_{Z}(\mathcal{A})\leq \max_{a_{i_{1} \cdots i_{m} } \ne0} \Biggl\{ { \prod_{j = 1}^{m} {r_{i_{j} }^{\frac{1}{m}} (\mathcal{A})} } \Biggr\} . $$

Case 2. If $\mathcal{A}$ is reducible. Let $\mathcal{T} =(t_{i_{1}i_{2} \cdots i_{m}})$, $t_{i_{1}i_{2} \cdots i_{m} }=1$ for all $1\leq i_{1},i_{2}, \ldots , i_{m} \leq n $. Then $\mathcal{A} + \epsilon\mathcal{T}$ is an irreducible nonnegative tensor for any chosen positive real number *ϵ*. Now we substitute $\mathcal{A} + \epsilon\mathcal{T}$ for $\mathcal{A}$, respectively, in the previous case. When $\epsilon \rightarrow0$, the result follows by the continuity of $\rho_{Z}(\mathcal {A} + \epsilon\mathcal{T} )$. □

By Theorem 3.1, a bound on the *Z*-spectral radius of a uniform hypergraph is obtained, we also compare the bound with the result in (1).

### Theorem 3.2

*Let* $\mathcal{H}$ *be a* *k*-*uniform hypergraph on* *n* *vertices with the degree sequence* $\Delta=d_{1} \geq\cdots\geq d_{n}=\delta$. *Then*

3 $$ \rho_{Z} (\mathcal{H}) \le \max_{\{i_{1},\ldots,i_{k} \} \in E(H)} \Biggl\{ {\prod_{j = 1}^{k} {d_{i_{j} }^{\frac{1}{k}} (\mathcal{A})} } \Biggr\} . $$

### Proof

Case 1. $\mathcal{A}(\mathcal{H})$ is irreducible. In this case, by Lemma 2.1, there exists a positive eigenvector corresponding to the spectral radius $\rho_{Z}(\mathcal{H})$. Then, by Theorem 3.1, we have

$$\rho_{Z} (\mathcal{H}) \le \max_{\{i_{1},\ldots,i_{k} \} \in E(H)} \Biggl\{ { \prod_{j = 1}^{k} {d_{i_{j} }^{\frac{1}{k}} (\mathcal{A})} } \Biggr\} . $$

Case 2. If $\mathcal{A}(\mathcal{H})$ is reducible. Let $\mathcal{T} =(t_{i_{1}i_{2} \cdots i_{k} })$, $t_{i_{1}i_{2} \cdots i_{k} }=1$, for all $1\leq i_{1},i_{2}, \ldots, i_{k} \leq n $. Then $\mathcal{A}(\mathcal{H}) + \epsilon\mathcal{T}$ is an irreducible nonnegative tensor for any chosen positive real number *ϵ*. Now we substitute $\mathcal {A}(\mathcal{H}) + \epsilon\mathcal{T}$ for $\mathcal{A}(\mathcal {H})$, respectively, in the previous case. When $\epsilon\rightarrow 0$, the result follows by the continuity of $\rho_{Z}(\mathcal{A}(\mathcal {H}) + \epsilon\mathcal{T} )$. □

### Remark

Obviously, we can get

$$\max_{\{i_{1},\ldots,i_{k} \} \in E(H)} \Biggl\{ {\prod_{j = 1}^{k} {d_{i_{j} }^{\frac{1}{k}} (\mathcal{A})} } \Biggr\} \leq\Delta. $$

That is to say, our bound in Theorem 3.2 is always better than the bound in (1).

We now show the efficiency of the new upper bound in Theorem 3.2 by the following examples.

### Example 1

Consider 3-uniform hypergraph $\mathcal{H}_{1}$ with a vertex set $V(\mathcal{H}_{1})=\{1,2,3,4,5,6,7\}$ and an edge set $E(\mathcal{H}_{1}) =\{e_{1}, e_{2} , e_{3}\}$, where $e_{1} = \{1, 2, 3\}$, $e_{2} = \{1, 4, 5\}$, $e_{3} = \{1, 6, 7\}$.

### Example 2

Consider 3-uniform hypergraph $\mathcal{H}_{2}$ with a vertex set $V(\mathcal{H}_{2})=\{1,2,3,4,5,6,7\}$ and an edge set $E(\mathcal{H}_{2}) =\{e_{1}, e_{2} , e_{3}\}$, where $e_{1} = \{1, 6, 7\}$, $e_{2} = \{2, 6, 7\}$, $e_{3} = \{3, 6, 7\}$.

From Table 1, we can find that bound (3) is always better than (1).

**Table 1:** Upper bounds for the hypergraphs $\mathcal{H}_{1}$ and $\mathcal{H}_{2}$

	(1)	(3)
$\mathcal{H}_{1}$	3	$3^{\frac{1}{3}}$
$\mathcal{H}_{2}$	3	$3^{\frac{2}{3}}$

## Conclusion

In this paper, we get a new bound for the *Z*-spectral radius of tensors. As applications, in terms of the degree sequence $d_{i}$, we obtain a new bound for the *Z*-spectral radius of hypergraphs, which is always better than the bound in [8]. We list two examples to show the efficiency of our new bound.
